# Resolution of Angina Pectoris and Improvement of the Coronary Flow Reserve after Ranolazine Treatment in a Woman with Isolated Impaired Coronary Microcirculation

**DOI:** 10.1155/2013/343027

**Published:** 2013-03-13

**Authors:** Alessandro Santoro, Vincenzo Schiano Lomoriello, Ciro Santoro, Riccardo Muscariello, Maurizio Galderisi

**Affiliations:** ^1^Cardioangiology with CCU, Department of Clinical and Experimental Medicine, Federico II University Hospital, 80131 Naples, Italy; ^2^Laboratory of Echocardiography, Cardioangiology with CCU, Department of Clinical and Experimental Medicine, Federico II University Hospital, 80131 Naples, Italy

## Abstract

In a 61-year-old woman with well controlled arterial hypertension, hypercholesterolemia, and smoke and suffering from recurrent angina pectoris despite angiographically normal epicardial coronary vessels and maximal therapy, the replacement of nitrates with novel antiangina drug ranolazine, after 6-month therapy, induced a complete relief of angina and a relevant rising of the transthoracic Doppler-derived coronary flow reserve (CFR). The present clinical case underlines therefore how in patients with chronic ischemic heart disease without epicardial coronary stenosis ranolazine can induce an improvement till the complete solution of the angina symptoms and a substantial increase of CFR as expression of the enhancement of the microvascular coronary function. The improvement of both symptoms and coronary microvascular function is strictly linked to the mechanism of action of the drug. Ranolazine induces in fact a reduction of the intracellular late sodium current that leads to a reduction of the intracellular calcium concentration thus producing a better myocardial diastolic relaxation process which in its turns enhances the myocardial perfusion. The ranolazine acts therefore as a lusitropic drug that improves the diastolic dysfunction and the segmental ischemia thus affecting one of the first steps of the ischemic cascade.

## 1. Introduction

Chronic angina pectoris represents a common impairing disease that involves limitations in the work activities and affects the individual quality of life [1]. Though the revascularization procedures are developing day-by-day, still a relevant number of patients (up to 20%) keep showing the angina symptoms even if they have been undergone a percutaneous coronary angioplasty intervention (PCI) and/or coronary artery bypass and are treated by standard therapy (beta blockers, calcium antagonists, and nitrites) with the maximal dosage [2]. Although several causes might be considered as responsible of this frequent treatment failure, coronary microvascular impairment is one of the main determinant taking part as well to the development of the angina symptoms. In addition, the activity of the standard therapy is based on hemodynamic effects which involve the reduction of myocardial oxygen consumption while novel mechanisms of action might be used to support and enhance the anti-ischemic effect. Ranolazine, an example of a new class of antiangina drugs, has shown a good outcome despite the absence of hemodynamic effects with an increasing number of clinical experiences that certify its value in the chronic ischemic heart disease [3–5].

## 2. Clinical Case

**Figure 1:**
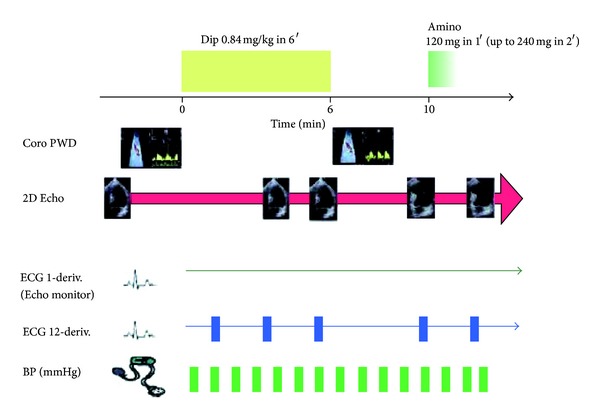
Scheme of “fast” protocol with dipyridamole for evaluating CFR and regional wall motion according to the recommendations of the European Association of Echocardiography [6].

**Figure 2:**
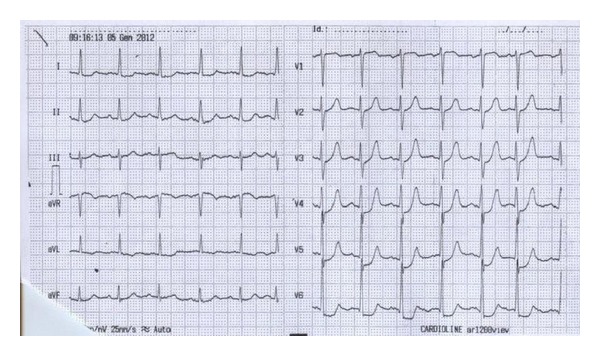
Surface ECG performed at the high-dose dipyridamole during stress echocardiography, showing a clear depression of the antero-septal ST segment. At the same time the patient referred angina pectoris.

**Figure 3:**
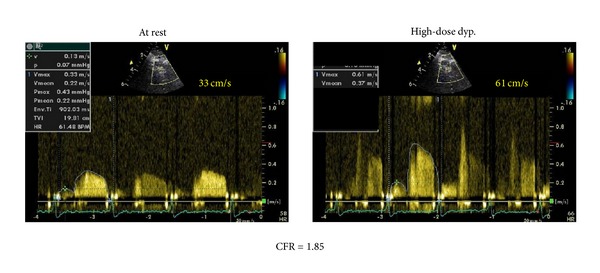
High-dose dipyridamole induced CFR during the same stress echocardiography of Figure 2.

**Figure 4:**
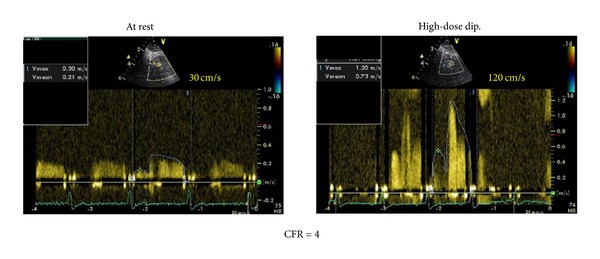
CFR evaluation after 6-month therapy with ranolazine.

**Figure 5:**
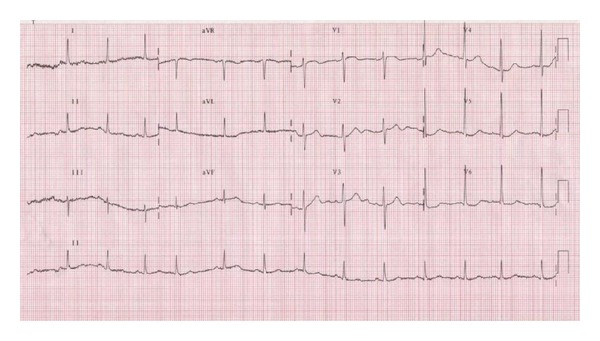
Surface ECG performed at high-dose dipyridamole during stress echocardiography after 6-month therapy with ranolazine. At the same time the patient was completely asymptomatic.

Woman, 61 years, affected by a long lasting, well controlled arterial systemic hypertension, hypercholesterolemia, and smoke (20 cigarettes daily). In the last 3 years she presented several angina episodes (5-6 per week). At June 2010 she underwent a coronary angiography showing the absence of significant stenosis of the epicardial coronary arteries. The patient was treated with bisoprolol 10 mg oid, valsartan 320 mg oid, isosorbide-5-mononitrate 60 mg oid, acetylsalicylic acid (ASA) 100 mg oid, and rosuvastatin 20 mg oid. Even after this therapy the symptoms kept affecting the patient. In December 2010, she underwent a pharmacologic stress echocardiography with dipyridamole (0.84 mg/Kg in 6 minutes, “fast” protocol) to evaluate at the same time the coronary flow reserve (CFR) and the regional wall motion according to the stress echo recommendations of the European Association of Echocardiography (Figure 1) [6]. In the presence of angina pectoris and significant repolarization phase abnormalities of the surface ECG (Figure 2) the stress echo showed an impaired CFR (<2) (Figure 3) without regional wall motion alteration. According to the results of the test an isolated microvascular coronary dysfunction was diagnosed (reduced CFR + normal regional wall motion) [6]. Accordingly, given that the symptoms were still present, it was decided to replace the isosorbide-5-mononitrate with ranolazine, for the first 2 weeks 375 mg bid followed by 500 mg bid. At the third month from the beginning of the therapy (March 2011) the patient referred a substantial reduction of the angina pectoris rate through the week (from 5-6 to 2 per week) and the complete resolution of the symptoms in May 2011. In June 2011 new pharmacologic stress echo was repeated. Again in the absence of regional wall motion abnormalities, the test showed a completely normal CFR with a relevant improvement in comparison with the previously performed test (from 1.85 to 4.00) (Figure 4). Of interest, the patient did not exhibit any kind of symptoms during and after the stress echo and the surface ECG was totally normal (Figure 5). The patient is yet under treatment with ranolazine (combined with bisoprolol, valsartan, ASA, and rosuvastatin) and she is completely free of symptoms of angina pectoris.

## 3. Discussion

The present clinical case underlines how in patients with chronic ischemic heart disease without epicardial coronary stenosis the replacement of nitrates with ranolazine can obtain (1) an improvement till the complete solution of the angina symptoms, (2) a relevant increase of CFR as expression of the enhancement of the microvascular coronary function.

The women affected by angina pectoris and stress-induced ischemia but free of obstructive coronary disease often show an isolated microvascular coronary dysfunction that brings not only a low quality of life but also an adverse prognosis for cardiovascular events during the follow-up [7].

The persistence of the angina though a maximal anti-ischemic therapy represents a therapeutic issue that needs to be faced. In the present clinical case ranolazine showed not only to be useful in reducing the frequency till the resolution of the angina episode but also in improving CFR substantially.

**Figure 6:**
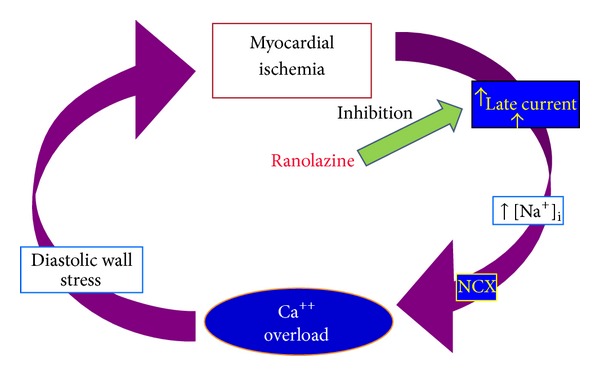
Mechanism of action of ranolazine. The pharmacologically induced reduction of the late sodium current ameliorates the myocardial diastolic relaxation by reducing the diastolic wall stress. This finally produces an improvement of segmental myocardial ischemia.

According to the recommendation of the European Association of Echocardiography [6], the stress echo with dipyridamole represents a very accurate test in order to distinguish an epicardial coronary stenosis from an isolated coronary microvascular dysfunction: the first one shows a reduction of the CFR with impairment of the regional wall motion and the latter shows a reduction of the CFR without regional wall motion abnormalities or even a supernormal wall motion. In our experience a reduced CFR + normal regional wall motion pointed out an impaired function of the coronary microcirculation, where a previous coronary angiography had already shown a coronary three free of stenosis. In this setting, after almost 6 months of therapy, ranolazine caused a relevant rising of the CFR that is strictly linked to its mechanism of action. Ranolazine induces a reduction of the intracellular late sodium current that leads to a reduction of the intracellular calcium concentration thus producing a diastolic relaxation improvement which in its turns enhances the myocardial perfusion [8]. The coronary flow is substantially greater during the diastolic phase of the cardiac cycle thus it is evident that the smaller vessels, the ones that make up the microvascular circulation, are the one that benefit more from the effect of the decompression caused by ranolazine during the myocardial relaxation process. The ranolazine acts therefore as a lusitropic drug that improves the diastolic dysfunction and the segmental ischemia thus affecting one of the first steps of the ischemic cascade (Figure 6). The results of the presented clinical case reflect the ones produced by a recently published clinical study where a 4-week therapy with ranolazine showed to be useful reducing the recurrence of the angina symptoms and improving the CFR assessed by cardiac magnetic resonance after adenosine infusion in a population of women without obstructive coronary disease [9].
